# Mesostigmata mite assemblages in stands developed on former agricultural land

**DOI:** 10.1007/s10493-026-01174-3

**Published:** 2026-08-24

**Authors:** Jacek Malica, Cezary K. Urbanowski, Emilia Pers-Kamczyc, Szymon Nowaczyński, Jacek Kamczyc

**Affiliations:** 1 Department of Game Management and Forest Protection, Faculty of Forestry and Wood Technology, Poznań University of Life Sciences, Wojska Polskiego 28, Poznań, 60-637 Poland; 2 Institute of Dendrology, Polish Academy of Sciences, Parkowa 5, Kórnik, 62-035 Poland; 3 Municipal Forests Unit in Poznań, Ku Dębinie 2, Poznań, 61-492 Poland

**Keywords:** Afforestation, Habitat differentiation, Soil fauna, Soil restoration, Tailings storage facility, Tree species effects

## Abstract

Afforestation of post-agricultural land plays a crucial role in restoring forest soil properties and initiating secondary succession processes driven by soil biota. In this study, we compared Mesostigmata communities in even-aged (21 y./o.) stands of *Alnus glutinosa*,* Betula pendula*,* Picea abies*,* Pinus sylvestris*, and *Tilia cordata* established on a former agricultural lands, and adjacent fallow field as a reference habitat, located near the Żelazny Most tailings storage facility (Lubin Forest District, Poland). Soil sampling was conducted on 18 study sites (5 tree species and fallow field × 3 plots) using steel corer (0–15 cm depth), consisting of 90 samples (5 samples from each plot). In total, 592 mesostigmatid mites representing 39 taxa and 15 families were recorded. Community structure differed among habitats, with *Alnus glutinosa* supporting the highest abundance and diversity, while *Tilia cordata* exhibited the lowest values. Deciduous stands generally hosted richer and more abundant assemblages than coniferous stands and the fallow field. The occurrence of both widespread generalists and highly specialized species highlights clear habitat differentiation and varying degrees of ecological specialization, reflecting differences in soil restoration processes among tree species.

## Introduction

In Europe afforest ation is used as part of its climate policy, treating it as a cost-effective tool for CO₂ sequestration that complements emission reduction efforts in sectors such as energy and industry. Economic analyses show that increasing forest cover, together with appropriate timber management practices (e.g., harvesting rotation and wood utilization), can significantly reduce the overall costs of achieving climate targets (Austin et al. 2020). However, the effectiveness of this strategy depends on its integration with other policy instruments and long-term forest management planning (Michetti and Rosa 2012). Afforestation of former agricultural land is one of the fundamental tasks of contemporary forest management (Fayet et al. 2022; Malica et al. 2022). Establishing stands in deforested areas that have been used for agricultural purposes for a long time is a highly demanding task and must be based on the appropriate selection of the stand’s species composition (Gorzelak 1996). The first generation of trees growing on such areas plays a key role in their restoration and in re-establishing the characteristics typical of forest soils. These include, among others, a lower pH, higher organic matter content, and an increased carbon-to-nitrogen ratio (Gorzelak 1996; Krawczyk 2014; Rosenzweig et al. 2016). This process is accompanied by the secondary succession of other organisms characteristic of forest habitats (Dunger et al. 2001; Ge et al.., 2019; Háněl 2008). The proper course of soil restoration is also driven by fungi, bacteria, nematodes, and soil microarthropods, which regulate the decomposition of dead organic matter (Biró et al. 1993; Huhta et al. 1991; Malica et al. 2024a, b; Neher et al. 2003; Neher and Barbercheck 2019; Urbanowski et al. 2021). Different tree species modify the properties of post-agricultural soils in distinct ways, influencing parameters such as pH, nutrient availability, and litter characteristics (Thomaes et al. 2012). Oak stands are often associated with higher pH and calcium accumulation, while pine stands tends to promote lower pH and different C/N dynamics (Malica et al. 2022). Consequently, the success and trajectory of land restoration strongly depend on tree species selection, as tree species shape organic matter turnover rates and long-term soil chemical development, thereby directing ecosystem recovery processes (Malica et al. 2024a, b).

In the present study, the response of the soil environment to the afforestation of post-agricultural land with different tree species was characterised using mites of the order Mesostigmata. Due to their position mainly as predators at the highest levels of the soil food webs and their high sensitivity to ecosystem changes, they are effective bioindicators (Walter and Proctor, 2013). Additionally, the structure of Mesostigmata assemblages is linked to the physicochemical properties of the soil, such as soil pH, soil water conditions, organic matter content and content of elements (Kamczyc et al., 2018, 2023; Malica et al. 2025). As numerous predators inhabiting both the litter layer and mineral soil, they regulate abundance of other soil organisms and thus indirectly reflect changes in their own abundance, which is sensitive to changes in soil environmental conditions (Kamczyc et al. 2017; Koehler 1997). Therefore, soil mites of the order Mesostigmata are considered good bioindicators of forest restoration on post-agricultural land (Delcourt et al. 2023; Malica et al. 2024a, b). Differences in abundance, species richness, and species diversity indicated variation in the effects of particular tree species on the soil environment, as well as on the rate and direction of secondary succession of mites (Delcourt et al. 2023).

Thus the main goal was to analyse Mesostigmata mite communities inhabiting even-aged *Alnus glutinosa* L., *Betula pendula* Roth., *Picea abies* (L.) H. Karst, *Pinus sylvestris* L., and *Tilia cordata* Mill. stands growing on rusty soils, and adjacent fallow field. We formulated the following hypotheses: (1) Mesostigmata mite assemblages differ among forest stands and the fallow field, with forest stands showing more advanced successional characteristics, including higher mite abundance, species richness, diversity, and a higher proportion of specialist taxa compared to the fallow field, reflecting increased habitat stability and development following reforestation (Crotty 2020; Insfrán Ortiz et al. 2023). (2) Differences among tree stands will be more strongly reflected in mite species composition and diversity-based metrics (species richness and diversity) than in total abundance. Mesostigmata community structure will significantly differentiate the studied stands, enabling the identification of stands representing more advanced successional stages and better post-reforestation conditions, thereby confirming their bioindicator value (Meehan et al. 2019; Malica et al. 2022).

## Materials and methods

### Study area, sampling and extraction

This study was conducted in a managed forest in the Lubin Forest District (SW Poland) near the Żelazny Most tailings storage facility, with the studied stand located 1,800 m from its edge (51.482115 °N, 16.180908 °E). Due to the immediate proximity of the tailings storage facility, the surrounding forests are subjected to significant industrial pressure (Gurwin et al. 2024). The climate of the region is temperate. Mean annual temperature reaches 8.8 °C and mean annual precipitation reaches 533 mm (Jakubczyk 2019). At the beginning of the study, we established research plots which meet the following criteria: (1) they were established on former agricultural land; (2) they are located on the same soil type; (3) the stands are of the same age; and (4) agricultural field is present in the vicinity.

**Fig. 1:**
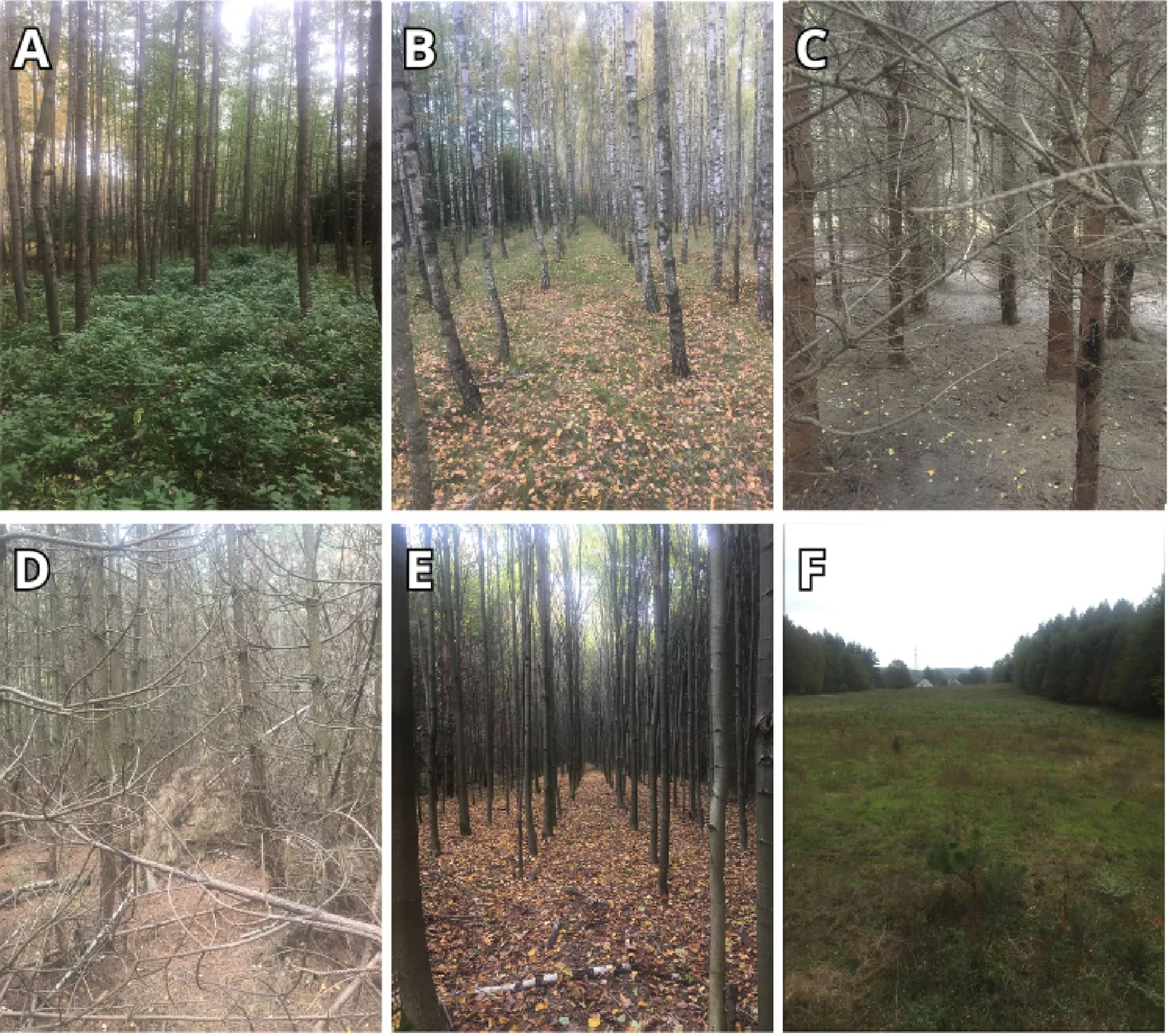
Studyplots on post-agricultural lands (October, 2022) in the Lubin Forest District: A– *Alnus glutinosa* stand, B– *Betula pendula* stand, C– *Picea abies* stand, D– *Pinus sylvestris* stand, E – *Tilia cordata* stand, F – fallow field (control)

Finally, we established 18 study sites (6 habitats × 3 plots each) in 21 y./o. alder (*Alnus glutinosa*), silver birch (*Betula pendula*), Norway spruce (*Picea abies*), Scots pine (*Pinus sylvestris*), and small-leaved lime (*Tilia cordata*) forests and fallow field as control plot (Fig. 1). All the stands studied formed a single, contiguous forest complex, adjacent to the fallow field under study. In an alder stand, the ground vegetation was dominated by *Urtica dioica* L., whereas in a birch stand and on the fallow field, the ground layer was dominated by grasses such as *Calamagrostis arundinacea* (L.) Roth. The total afforested study area covered 2.62 ha, with each stand occupying approximately 0.40 ha. A fallow field adjacent to the forest stands was included as a control habitat. Samples were collected at a distance greater than the stand height to minimize edge effects. The site represents post-arable land left fallow for more than one growing season after wheat cultivation. All plots were located on Cambisols. — acc. to IUSS Working Group WRB, (2022). Plots within each habitat were spaced at a minimum distance of 15 m from one another to avoid spatial autocorrelation. From each plot, five soil cores were collected using a metal corer (ø = 5 cm, depth 0–15 cm), resulting in a total of 90 soil samples (6 habitats × 15 samples per habitat) on October 14, 2022.

Soil samples were transported to the laboratory in a portable cooler and processed using a Berlese-Tullgren apparatus, which consisted of a funnel, a 40 W light bulb, a 2 mm mesh strainer, and containers filled with 75% ethanol. Soil fauna were extracted from the samples for at least seven days, until the samples were completely dry. Mesostigmatid mites were selected from the ethanol solution under a stereomicroscope and mounted in Hoyer’s medium on microscope slides. Mite classification was conducted using a Zeiss Axio Scope.A1 microscope. All mites were classified to species level, or to a higher taxonomic unit when species-level identification was not possible, following standard identification keys (Gwiazdowicz 2007; Karg 1971, 1993; Masan 2003; Micherdziński 1969).

### Data analysis

Abundance, species richness, and diversity were calculated per sample. Mean values and standard errors (SE) were computed for each metric across habitat types. Abundance was defined as the number of individuals per sample. Species richness corresponded to the total number of distinct taxa per sample. Shannon-Wiener diversity index (H’) was calculated using the vegan package *(diversity(comm*,* index = “shannon”))*, integrating species richness and evenness: H’ = *-* Σpi × ln(pi), where pi is the proportion of individuals of the ith species. Species richness was calculated with *specnumber(comm)*.

Differences in abundance, richness, and diversity across habitat types were assessed using One -Way ANOVA, followed by Tukey’s HSD test (*TukeyHSD()*) for pairwise comparisons. Species–habitat interactions were visualized with bipartite web plots (*plotweb()*,* specieslevel()*), and Species Specificity Index (SSI) quantified habitat specialization (0 = ubiquitous, 1 = restricted). To estimate true species richness accounting for rare taxa, the Chao1 estimator was applied using the*vegan* and*iNEXT* packages. This non-parametric method provides observed richness (the actual number of species recorded in the samples), as well as asymptotic richness estimate (Chao1; the estimated true number of species, accounting for singletons and doubletons). The comparison of observed and estimated richness was used to evaluate sampling completeness and potential underrepresentation of rare taxa across habitat types. All analyses were conducted in R (R version 4.1.2; R Core Team R: A Language and Environment for Statistical Computing; available online: https://www.r-project.org/; iNext: Hsieh et al. 2016; vegan: Oksanenet al. 2019.)

## Results

In the conducted study, 592 mesostigmatid mites were collected and classified to 15 families and 39 taxa. The most abundant family across all samples was Parasitidae, represented by 118 individuals (19.93% of all mites), while the most abundant species was *Oodinychus ovalis* (Kramer), with 94 individuals recorded.

**Fig. 2:**
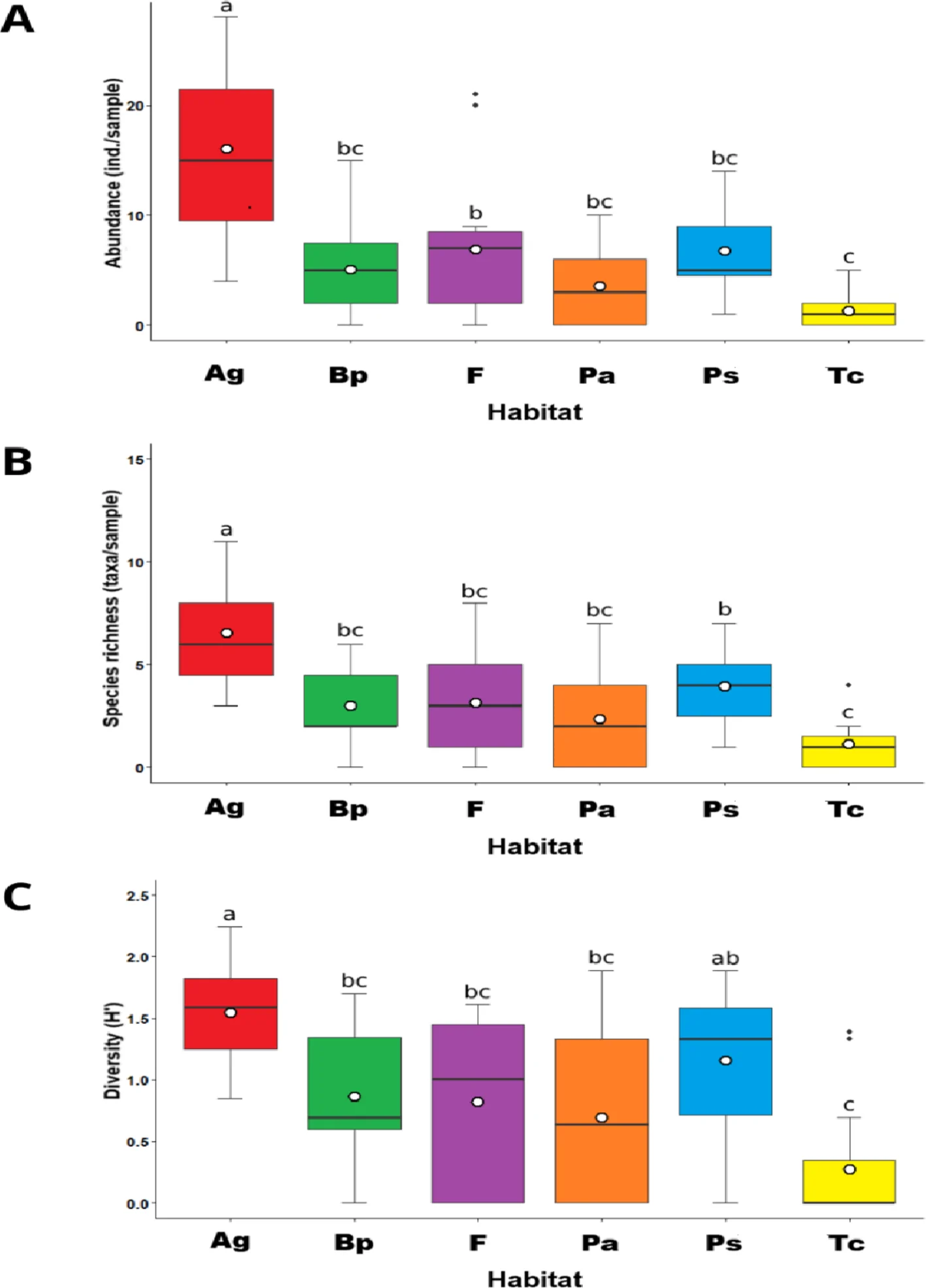
Meanabundance (A), species richness (B) anddiversity (C) of Mesostigmata mite communities. White dots indicate mean values, black dots are outliers, black lines are medians and while bars indicate interquartile range. Letters above the bars indicate post-hoc test results; bars sharing the same letters are not significantly different from each other. Abbreviations: Ag – *Alnus glutinosa*, Bp – *Betula pendula*, F – Fallow field, Pa – *Picea abies*, Ps – *Pinus sylvestris*, Tc – *Tilia cordata*

Habitat type had a significant effect on abundance, species richness, and diversity, with all ANOVA models showing highly significant differences among groups (*p* < 0.001) (Appendix A, Table 1). Overall, deciduous stands, particularly *Alnus glutinosa*, hosted more abundant and diverse mesostigmatid mite assemblages compared to conifers and open field habitats. Mesostigmatid mite communities varied notably among tree habitat types. *Alnus glutinosa* stand has the highest mean abundance (16.00 ± 4.95), species richness (6.53 ± 0.67), and diversity (1.55 ± 0.10), whereas *Tilia cordata* stand exhibited the lowest values across allmite assemblages characteristics (abundance: 1.27 ± 0.55; richness: 1.13 ± 0.35; diversity:0.27 ± 0.10). Intermediate values were observed for *Betula pendula* stand, fallow field, *Picea abies*, and *Pinus sylvestris* stands, with mean abundances ranging from 3.53 to 6.87, species richness from 2.33 to 3.93, and diversity from0.69 to1.15 (Fig. 2; Table 1).

**Fig. 3:**
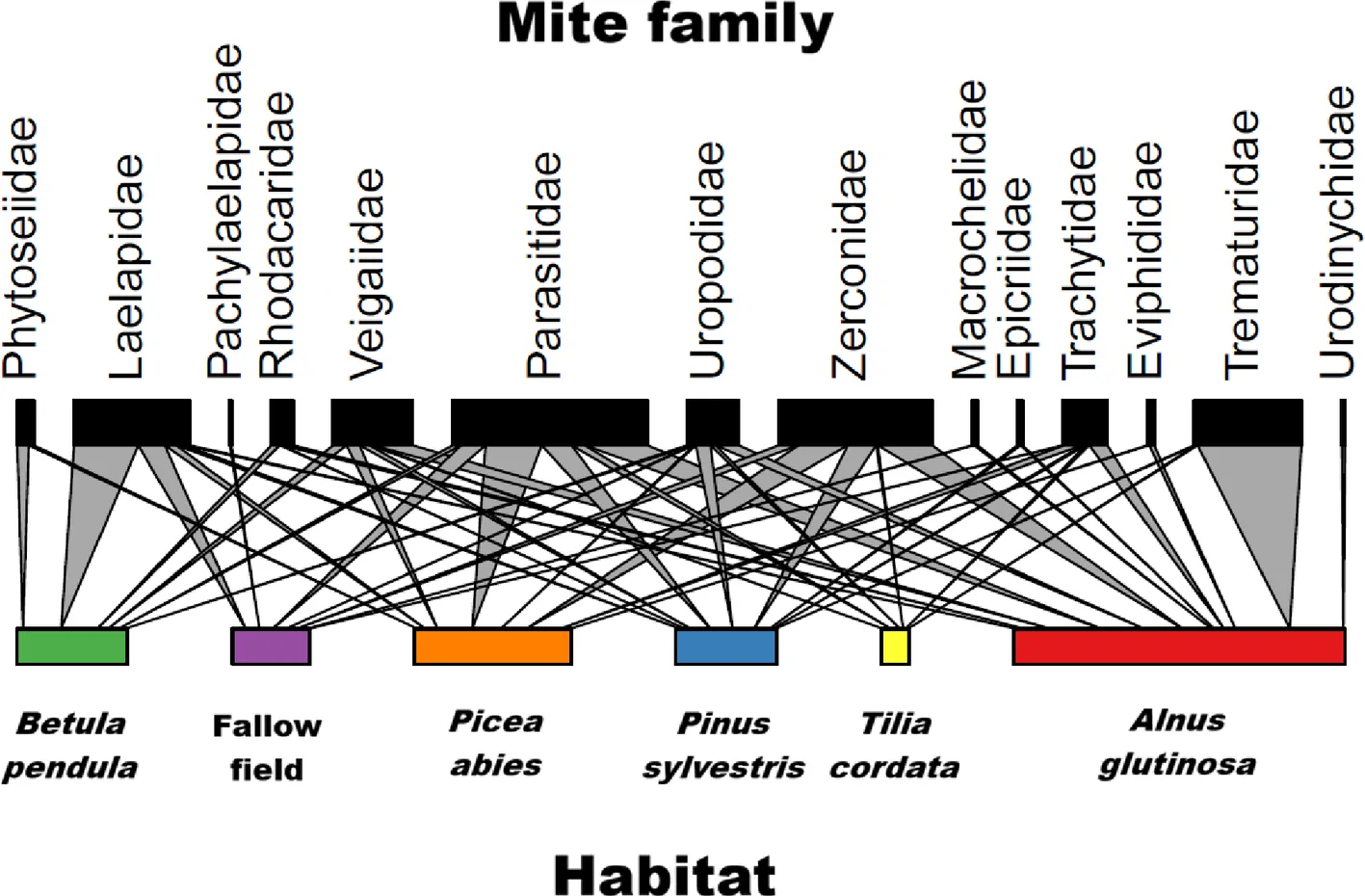
Co-occurrence network for bipartite relationships between mesostigmatid mite family (upper boxes) and habitat (lower boxes). Boxes are proportional to total mite abundance, whereas ribbon width is proportional to the co-occurrence

The habitat distribution and specificity of mesostigmatid mite families varied markedly among studied stands. Parasitidae and Uropodidae occurred in all surveyed habitats (proportion of habitats = 1.0), with Parasitidae showing the highest species-level specialization index (d’ = 1.71) and moderate Species Specificity Index (SSI = 0.28), while Uropodidae had a lower d’ (0.47) and SSI (0.097). Families such as Laelapidae, Trachytidae, Veigaiidae, and Zerconidae were present in most habitats (proportion = 0.83), with Laelapidae exhibiting particularly high habitat association (d’ = 1.09) and SSI (0.42). Other families, including Epicriidae, Eviphididae, Macrochelidae, Phytoseiidae, Rhodacaridae, and Trematuridae, showed intermediate habitat occurrence (proportion 0.33–0.50) and moderate d’ and SSI values. Pachylaelapidae and Urodinychidae were the most specialized, occurring in only a single habitat each (proportion = 0.17) with low to moderate specificity indices (d’ = 0.021 and 0.010, respectively). Overall, Parasitidae and Laelapidae were generalist families widely distributed across habitats, whereas Pachylaelapidae and Urodinychidae were highly habitat-specific. The most abundant family in the *Alnus glutinosa* stand was Trematuridae (26.3% of all individuals in this stand). In the case of *Betula pendula*,* Pinus sylvestris*, and *Tilia cordata* stands, it was Parasitidae. In the *Picea abies* stand and on the fallow field, it was Laelapidae (Fig. 3; Table 2).

**Fig. 4:**
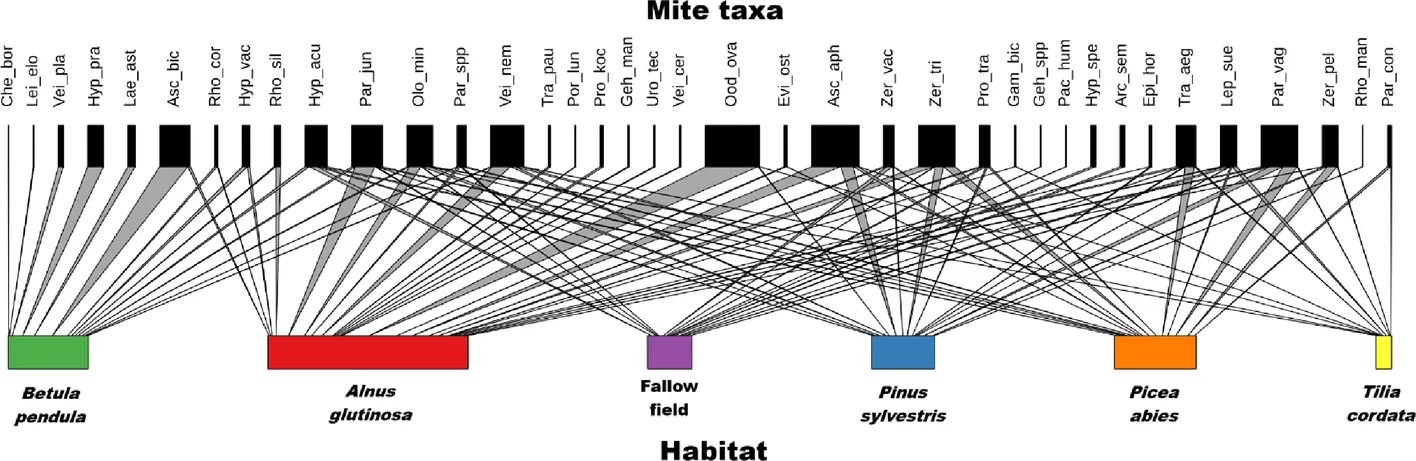
Co-occurrence network for bipartite relationships between mesostigmatid mite taxa (upper boxes) and collection Habitat (lower boxes). Boxes are proportional to total mite abundance, whereas ribbon width is proportional to the co-occurrence. For abbreviations of mite taxa see Table 2 in Appendix A

Overall, the data highlight a clear distinction between habitat specialists and generalists among the mesostigmatid mites (Fig. 4; Table 3). *Oodinychus ovalis* was the most abundant species in *Alnus glutinosa* stand, *Paragamasus vagabundus* (Karg) in *Betula pendula* and *Pinus sylvestris* stands, whereas *Leptogamasus suecicus* Trägårdh was most abundant in *Picea abies* and *Tilia cordata* stands (Appendix A, Table 2). Among the studied mesostigmatid mites, clear differences in habitat specialization were observed. A group of specialist species occurred exclusively in a single habitat, including *Arctoseius semiscissus* (Berlese), *Cheiroseius borealis* (Berlese), *Geholaspis mandibularis* (Berlese), *Hypoaspis praesternalis* Willmann, *Laelaspis astronomica* (Koch), *Leioseius elongatus* Evans, *Pachyseius humeralis* Berlese, *Porrhostaspis lunulata* Müller, *Prozercon kochi* Sellnick, *Trachytes pauperior* Berlese, *Urodiaspis tecta* Kramer, *Veigaia cerva* Kramer, and *V. planicola* (Berlese), all of which exhibit a Species Specificity Index (SSI) of 1, indicating strong habitat specialization. In contrast, generalist species such as *Olodiscus minima* (Kramer) (all habitats, SSI = 0.41), *Leptogamasus suecicus*, *Paragamasus vagabundus*, *Trachytes aegrota* (Koch), and *Veigaia nemorensis* (habitat proportion 0.83, SSI 0.34–0.49) were widely distributed across multiple habitats. Species occurring in an intermediate number of habitats showed moderate specificity (SSI 0.35–0.74), indicating partial habitat preference (Appendix A, Table 3).

**Fig. 5:**
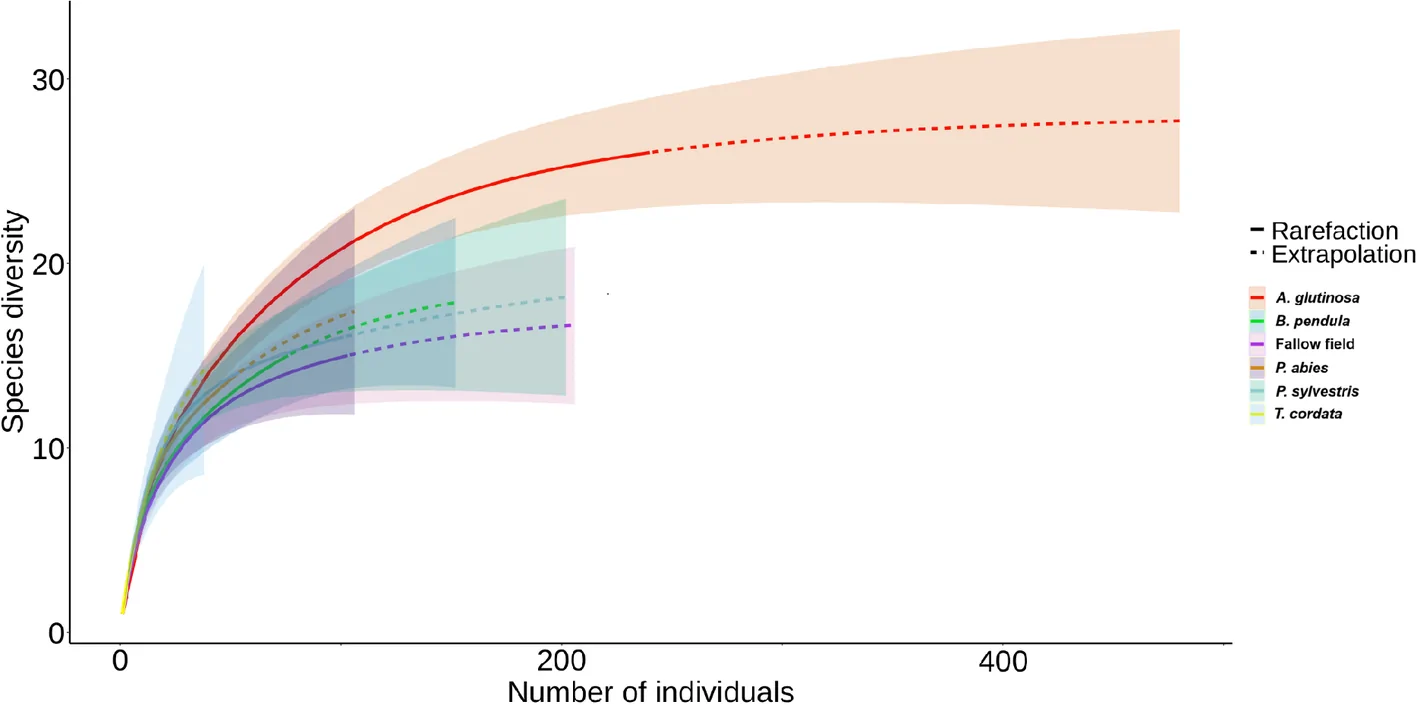
Rarefaction and extrapolation curves showing species diversity as a function of the number of individuals sampled among different habitats (The estimated richness of mite taxa in a community based on the Chao etimates, type = 1). Shaded areas indicate confidence intervals

The rarefaction and extrapolation analysis revealed that species richness increases with the number of individuals, but the rate of increase gradually levels off. The highest estimated richness is observed for *Alnus glutinosa* stand, while other species (e.g., *Betula pendula* and *Pinus sylvestris*) reach lower values and plateau more quickly. This suggests that sampling is nearly complete for most mite taxa, although additional sampling for *Alnus glutinosa* stand may still reveal more mite species (Fig. 5).

**Table 1:** Pairwise comparisons of mite abundance, species richness, and diversity among studied habitats based on Tukey’s HSD post hoc test, showing estimated differences, 95% confidence intervals (LCL–UCL), p-values. Abbreviations of variants: Ag – *Alnus glutinosa*, Bp – *Betula pendula*, F – Fallow field, Pa – *Picea abies*, Ps – *Pinus sylvestris*, Tc – *Tilia cordata*

Parameter		Comparison	Difference	95% CI (LCL–UCL)	*p*-value
Abundance		Ag – Bp	10.93	5.55–16.32	< 0.001
		Ag – F	9.13	3.75–14.52	< 0.001
		Ag – Pa	12.47	7.08–17.85	< 0.001
		Ag – Ps	9.27	3.88–14.65	< 0.001
		Ag – Tc	14.73	9.35–20.12	< 0.001
		F – Tc	5.60	0.22–10.98	0.0366
		Ps – Tc	5.47	0.08–10.85	0.0444
Species richness		Ag – Bp	3.53	1.29–5.78	< 0.001
		Ag – F	3.40	1.15–5.65	< 0.001
		Ag – Pa	4.20	1.95–6.45	< 0.001
		Ag – Ps	2.60	0.35–4.85	0.0137
		Ag – Tc	5.40	3.15–7.65	< 0.001
		Ps – Tc	2.80	0.55–5.05	0.0061
Species diversity		Ag – Bp	0.68	0.06–1.30	0.0226
		Ag – F	0.73	0.11–1.34	0.0122
		Ag – Pa	0.85	0.24–1.47	0.0017
		Ag – Tc	1.27	0.65–1.89	< 0.001
		Ps – Tc	0.88	0.26–1.50	0.0011

**Table 2:** Abundance of Mesostigmata mites at the family level in different habitats studied. The values represent the total number of individuals recorded across all samples

No.	Family	Alnus glutinosa	Betula pendula	Fallow field	Picea abies	Pinus sylvestris	Tilia cordata	Total
1	Ascidae	39	15	36	6	6	2	104
2	Epicriidae	1	2	-	-	-	-	3
3	Eviphididae	3	-	-	-	1	-	4
4	Laelapidae	3	5	39	16	7	-	70
5	Macrochelidae	2	-	-	1	-	-	3
6	Pachylaelapidae	-	-	-	1	-	-	1
7	Parasitidae	38	18	5	15	34	8	118
8	Phytoseiidae	-	-	7	-	3	-	10
9	Rhodacaridae	7	1	5	-	-	-	13
10	Trachytidae	11	2	-	1	11	2	27
11	Trematuridae	63	1	-	-	-	1	65
12	Urodinychidae	2	-	-	-	-	-	2
13	Uropodidae	14	9	3	1	1	3	31
14	Veigaiidae	24	6	8	-	8	2	48
15	Zerconidae	33	17	-	12	30	1	93
	**Total**	**240**	**76**	**103**	**53**	**101**	**19**	**592**

## Discussion

Based on all analyzed parameters of Mesostigmata mite assemblages, *Alnus glutinosa* stands appear to provide the most favorable conditions for these organisms. Mite communities inhabiting these stands were the most abundant and the most diverse in terms of species richness. Statistically, only *Pinus sylvestris* stand did not differ significantly in community diversity from *Alnus glutinosa* stand. Concurrently, the lowest values of abundance, species richness, and diversity were observed in *Tilia cordata* stand, indicating that they were the least colonized by the studied mites. Considering that the fallow field (control) exhibited intermediate values of these parameters, the first hypothesis was not supported. According to this hypothesis, a treeless area with poor vegetation cover was expected to harbor the most impoverished Mesostigmata mite communities. Similarly, the second hypothesis was rejected, as differences in abundance, species richness, and diversity were comparable among the studied habitats.

Bazargaliyeva et al. (2024) demonstrated that *Alnus glutinosa* stands are associated with strongly moist habitats and a relatively diverse floristic composition, although in some regions the species occurs locally rarely and its distribution is declining due to anthropogenic pressures and limited natural regeneration. On former arable lands, *Alnus glutinosa* quickly improves soil conditions through its symbiosis with nitrogen-fixing bacteria, leading to increased soil fertility. Its easily decomposable, nitrogen-rich litter intensifies the activity of the soil biota, accelerating nutrient cycling and the release of nutrients. Additionally, the abundance of soil organisms (e.g., earthworms) increases, improving soil structure and aeration. As a result, soils under alder become more nutrient-rich, biologically active, and favorable for further plant succession (Fremstad 1983; Zitzer and Dawson 1992). In black alder stands developing on post-agricultural soils, the herb layer is characterized by a mix of species typical of wet habitats and fast-growing, competitive plants that take advantage of fertile soil, such as common nettle (*Urtica dioica* L.) and grasses. The recovery of the forest herb layer is faster in areas that are wet, fertile, and located close to old forests, which provide sources of seeds and young plants (Orczewska 2010). Finally, abundant understory vegetation promotes soil fauna because it provides litter and organic matter that serve as food and shelter for soil organisms, while also improving soil moisture, structure, and microhabitat conditions (Frouz 2008).

Linden (*Tilia* spp.) in deciduous forests has a moderate effect on the soil – improving its chemistry mainly in the surface layer, but in mixed stands this effect is weakly visible. However, when linden dominates the canopy, it strongly shades the understory, leading to a decline in plant species diversity and a shift toward shade-tolerant species, thereby reducing the functional diversity of the ecosystem (Langenbruch et al. 2012). Linden leaves decompose relatively quickly, but their effect on the soil is mostly superficial, which may not promote significant changes in the soil environment or the accumulation of litter that could potentially be colonized by larger aggregations of mites (De Jaegere et al. 2016; Urbanowski et al. 2021). This may explain the low abundance, species richness, and diversity of Mesostigmata mites in the studied small-leaved linden (*Tilia cordata*) stand.

The most dominant family in the *Alnus glutinosa* stand, Trematuridae, belongs to the suborder Uropodina and primarily inhabits forest habitats rich in dead organic matter. They prefer moderately moist forest litter and decayed wood (Błoszyk 2008). It is worth noting that the family Zerconidae was absent only from the fallow field, further confirming its role as a bioindicator of forest habitats (Malica et al. 2024a, b). In contrast, species belonging to Laelapidae and Ascidae were relatively abundant in the fallow field, with their high proportion being characteristic of agrocenoses (Malica et al. 2025).

Two species (*Trachytes aegrota* and *Leptogamasus suecicus*) were recorded in all habitats except the fallow field, which may indicate their potential use as bioindicators of the very early stage of forest succession on post-agricultural land. *Trachytes aegrota* is a common, eurytopic species within the Uropodina, associated both with dead-wood microhabitats and the litter of various types of deciduous forests (especially beech and oak), and occurring in moist pine forests, as well as in post-industrial areas (Napierała et al. 2009; Skorupski et al. 2013). In comparison, other Uropodina such as *Trachytes pauperior* and *Urodiaspis tecta* were recorded only in stand of *Alnus glutinosa*, whereas *Olodiscus minima* was found in all investigated habitats. Another species occurring in forest stands but not in the fallow field was *Leptogamasus suecicus*, which is generally also described from degraded and agricultural soils (Niedbała et al. 1982; Skorupski et al. 2013).

Due to the potential of black alder (*Alnus glutinosa*) to create favorable soil habitat conditions, special attention should be paid to this species when determining the tree species composition of afforestation on post-agricultural lands. However, its specific habitat requirements must also be taken into account. At the same time, caution is advised when establishingpure stands of small-leaved lime (*Tilia cordata*), due to the potential limitations they may impose on the rate of succession of certain soil organisms. The diverse effects of different tree species on this process suggest the need to create mixed-species stands on post-agricultural lands.

## Data Availability

We declare all data is being provided within this manuscript.

## Appendix A

**Table 3:** Results of one-way ANOVA testing the effect of tree species on abundance, species richness, and diversity. Significant differences among tree groups were detected for all response variables (*p* < 0.001)

Variable	Factor	Df	Sum Sq	Mean Sq	F value	Pr(> F)	Significance
Abundance	tree	5	1930	385.9	15.11	1.45e-10	***
	Residuals	84	2146	25.6	—	—	—
Species richness	tree	5	248.9	49.77	11.2	2.76e-08	***
	Residuals	84	373.5	4.45	—	—	—
Diversity	tree	5	13.88	2.777	8.217	2.36e-06	***
	Residuals	84	28.39	0.3379	—	—	—

**Table 4:** Abundance of Mesostigmata mites taxa in different forest habitats in Lubin Forest District. The values represent the total number of individuals recorded across all samples

No.	Taxa	Abbr.	Alnus glutinosa	Betula pendula	Fallow field	Picea abies	Pinus sylvestris	Tilia cordata	Total	Proportional abundance (%)
1	*Amblyseius* spp.	Amb_spp	-	-	7	-	3	-	10	1.69
2	*Arctoseius semiscissus* (Berlese, 1892*)*	Arc_sem	-	-	-	6	-	-	6	1.01
3	*Asca aphidioides* (Linneaus, 1758)	Asc_aph	35	15	-	-	6	1	57	9.63
4	*Asca bicornis* (Canestrini and Fanzago, 1877)	Asc_bic	3	-	34	-	-	-	37	6.25
5	*Cheiroseius borealis* (Berlese, 1904)	Che_bor	-	-	1	-	-	-	1	0.17
6	*Epicriopsis horridus* Kramer, 1876	Epi_hor	1	2	-	-	-	-	3	0.51
7	*Eviphis ostrinus* (C.L.Koch, 1836)	Evi_ost	3	-	-	-	1	-	4	0.68
8	*Gamasellodes bicolor* (Berlese, 1918)	Gam_bic	1	-	-	-	-	1	2	0.34
9	*Geholaspis mandibularis* (Berlese, 1904)	Geh_man	2	-	-	-	-	-	2	0.34
10	*Geholaspis* spp.	Geh_spp	-	-	-	1	-	-	1	0.17
11	*Hypoaspis aculeifer* (Canestrini, 1884)	Hyp_acu	-	5	5	10	7	-	27	4.56
12	*Hypoaspis praesternalis* Willmann, 1949	Hyp_pra	-	-	19	-	-	-	19	3.21
13	*Hypoaspis vacua* (Michael, 1891)	Hyp_sp	-	-	-	6	-	-	6	1.01
14	*Hypoaspis* spp.	Hyp_vac	3	-	6	-	-	-	9	1.52
15	*Laelaspis astronomica* (C.L.Koch, 1839)	Lae_ast	-	-	9	-	-	-	9	1.52
16	*Leioseius elongatus* Evans, 1958	Lei_elo	-	-	1	-	-	-	1	0.17
17	*Leptogamasus suecicus* Trägårdh, 1936	Lep_sue	4	1	-	7	2	6	20	3.38
18	*Olodiscus minima* (Kramer, 1882)	Olo_min	14	9	3	1	1	3	31	5.24
19	*Oodinychus ovalis* (C.L. Koch, 1839)	Ood_ova	63	1	-	-	-	1	65	10.98
20	*Pachyseius humeralis* Berlese, 1910	Pac_hum	-	-	-	1	-	-	1	0.17
21	*Paragamasus conus* (Karg 1971)	Par_con	-	-	-	-	4	1	5	0.84
22	*Paragamasus jugincola* (Athias-Henriot,1967)	Par_jug	24	1	4	2	6	-	37	6.25
23	*Paragamasus* spp.	Par_spp	3	-	1	2	5	-	11	1.86
24	*Paragamasus vagabundus* (Karg, 1968)	Par_vag	6	16	-	4	17	1	44	7.43
25	*Porrhostaspis lunulata* Müller, 1859	Por_lun	1	-	-	-	-	-	1	0.17
26	*Prozercon kochi* Sellnick, 1943	Pro_koc	4	-	-	-	-	-	4	0.68
27	*Prozercon traegardhi* (Halbert, 1923)	Pro_tra	4	2	-	4	3	-	13	2.20
28	*Rhodacarus coronatus* Berlese, 1920	Rho_cor	1	-	3	-	-	-	4	0.68
29	*Rhodacarus mandibularis* Berlese, 1920	Rho_man	-	1	-	-	-	-	1	0.17
30	*Rhodacarellus silesiacus* Willmann, 1936	Rho_sil	6	-	2	-	-	-	8	1.35
31	*Trachytes aegrota* (C.L.Koch, 1841)	Tra_aeg	8	2	-	1	11	2	24	4.05
32	*Trachytes pauperior* (Berlese, 1914)	Tra_pau	3	-	-	-	-	-	3	0.51
33	*Urodiaspis tecta* Kramer, 1876	Uro_tec	2	-	-	-	-	-	2	0.34
34	*Veigaia cerva* (Kramer, 1876)	Vei_cer	2	-	-	-	-	-	2	0.34
35	*Veigaia nemorensis* (C.L.Koch, 1839)	Vei_nem	22	6	2	-	8	2	40	6.76
36	*Veigaia planicola* (Berlese, 1892)	Vei_pla	-	-	6	-	-	-	6	1.01
37	*Zercon peltatus* C.L. Koch, 1836	Zer_pel	2	4	-	-	12	1	19	3.21
38	*Zercon triangularis* C.L. Koch, 1836	Zer_tri	19	10	-	1	14	-	44	7.43
39	*Zercon vacuus* C.L. Koch, 1839	Zer_vac	4	1	-	7	1	-	13	2.20
	**Total**		**240**	**76**	**103**	**53**	**101**	**19**	**592**	**100**

**Table 5:** Number of habitats, proportion of occupied habitats, dispersion index (d), and species specificity index for individual mite species

No.	Species	Abbr.	Number of habitats	Proportion of habitats	Index d	Species Specificity Index
1	*Amblyseius* spp.	Amb_spp	2	0.333	0.273	0.704
2	*Arctoseius semiscissus* (Berlese, 1892)	Arc_sem	1	0.167	0.518	1.000
3	*Asca aphidioides* (Linneaus, 1758)	Asc_aph	4	0.667	0.163	0.591
4	*Asca bicornis* (Canestrini and Fanzago, 1877)	Asc_bic	2	0.333	0.504	0.906
5	*Cheiroseius borealis* (Berlese, 1904)	Che_bor	1	0.167	0.154	1.000
6	*Epicriopsis horridus* Kramer, 1876	Epi_hor	2	0.333	0.134	0.683
7	*Eviphis ostrinus* (C.L.Koch, 1836)	Evi_ost	2	0.333	0.068	0.742
8	*Gamasellodes bicolor* (Berlese, 1918)	Gam_bic	2	0.333	0.167	0.633
9	*Geholaspis mandibularis* (Berlese, 1904)	Geh_man	1	0.167	0.053	1.000
10	*Geholaspis* spp.	Geh_spp	1	0.167	0.276	1.000
11	*Hypoaspis aculeifer* (Canestrini, 1884)	Hyp_acu	4	0.667	0.230	0.357
12	*Hypoaspis praesternalis* Willmann, 1949	Hyp_pra	1	0.167	0.507	1.000
13	*Hypoaspis vacua* (Michael, 1891)	Hyp_spe	1	0.167	0.518	1.000
14	*Hypoaspis* spp.	Hyp_vac	2	0.333	0.188	0.683
15	*Laelaspis astronomica* (C.L.Koch, 1839)	Lae_ast	1	0.167	0.410	1.000
16	*Leioseius elongatus* Evans, 1958	Lei_elo	1	0.167	0.154	1.000
17	*Leptogamasus suecicus* Trägårdh, 1936	Lep_sue	5	0.833	0.265	0.344
18	*Olodiscus minima* (Kramer, 1882)	Olo_min	6	1.000	0.084	0.413
19	*Oodinychus ovalis* (C.L. Koch, 1839)	Ood_ova	3	0.500	0.362	0.963
20	*Pachyseius humeralis* Berlese, 1910	Pac_hum	1	0.167	0.276	1.000
21	*Paragamasus conus* (Karg 1971)	Par_con	2	0.333	0.315	0.785
22	*Paragamasus jugincola* (Athias-Henriot,1967)	Par_jun	5	0.833	0.063	0.596
23	*Paragamasus* spp.	Par_spp	4	0.667	0.093	0.432
24	*Paragamasus vagabundus* (Karg, 1968)	Par_vag	5	0.833	0.207	0.413
25	*Porrhostaspis lunulata* Müller, 1859	Por_lun	1	0.167	0.000	1.000
26	*Prozercon kochi* Sellnick, 1943	Pro_koc	1	0.167	0.141	1.000
27	*Prozercon traegardhi* (Halbert, 1923)	Pro_tra	4	0.667	0.096	0.346
28	*Rhodacarus coronatus* Berlese, 1920	Rho_cor	2	0.333	0.156	0.742
29	*Rhodacarus mandibularis* Berlese, 1920	Rho_man	1	0.167	0.210	1.000
30	*Rhodacarellus silesiacus* Willmann, 1936	Rho_sil	2	0.333	0.116	0.742
31	*Trachytes aegrota* (C.L.Koch, 1841)	Tra_aeg	5	0.833	0.124	0.452
32	*Trachytes pauperior* (Berlese, 1914)	Tra_pau	1	0.167	0.108	1.000
33	*Urodiaspis tecta* Kramer, 1876	Uro_tec	1	0.167	0.053	1.000
34	*Veigaia cerva* (Kramer, 1876)	Vei_cer	1	0.167	0.053	1.000
35	*Veigaia nemorensis* (C.L.Koch, 1839)	Vei_nem	5	0.833	0.067	0.494
36	*Veigaia planicola* (Berlese, 1892)	Vei_pla	1	0.167	0.371	1.000
37	*Zercon peltatus* C.L. Koch, 1836	Zer_pel	4	0.667	0.235	0.590
38	*Zercon triangularis* C.L. Koch, 1836	Zer_tri	4	0.667	0.124	0.456
39	*Zercon vacuus* C.L. Koch, 1839	Zer_vac	4	0.667	0.199	0.525
